# Tanshinone IIA reduces SW837 colorectal cancer cell viability via the promotion of mitochondrial fission by activating JNK-Mff signaling pathways

**DOI:** 10.1186/s12860-018-0174-z

**Published:** 2018-09-25

**Authors:** Sayilaxi Jieensinue, Hong Zhu, Guangcheng Li, Keli Dong, Meiting Liang, Yayue Li

**Affiliations:** Department of Traditional Chinese Medicine, Central South University Third Xiangya Hospital, Changsha, 410013 China

**Keywords:** Tanshinone IIA, Mitochondrial fission, JNK-Mff pathways, Mitochondrial injury, Colorectal cancer

## Abstract

**Background:**

Mitochondrial homeostasis has been increasingly viewed as a potential target of cancer therapy, and mitochondrial fission is a novel regulator of mitochondrial function and apoptosis. The aim of our study was to determine the detailed role of mitochondrial fission in SW837 colorectal cancer cell viability, mobility and proliferation. In addition, the current study also investigated the therapeutic impact of Tanshinone IIA (Tan IIA), a type of anticancer adjuvant drug, on cancer mitochondrial homeostasis.

**Results:**

The results of our data illustrated that Tan IIA promoted SW837 cell death, impaired cell migration and mediated cancer proliferation arrest in a dose-dependent manner. Functional investigation exhibited that Tan IIA treatment evoked mitochondrial injury, as witnessed by mitochondrial ROS overproduction, mitochondrial potential collapse, antioxidant factor downregulation, mitochondrial pro-apoptotic protein upregulation, and caspase-9-dependent apoptotic pathway activation. Furthermore, we confirmed that Tan IIA mediated mitochondrial damage by activating mitochondrial fission, and the inhibition of mitochondrial fission abrogated the proapoptotic effects of Tan IIA on SW837 cells. To this end, our results demonstrated that Tan IIA modulated mitochondrial fission via the JNK-Mff pathways. The blockade of the JNK-Mff axis inhibited Tan IIA-mediated mitochondrial fission and promoted the survival of SW837 cells.

**Conclusions:**

Altogether, our results identified mitochondrial fission as a new potential target to control cancer viability, mobility and proliferation. Furthermore, our findings demonstrate that Tan IIA is an effective drug to treat colorectal cancer by activating JNK-Mff-mitochondrial fission pathways.

**Electronic supplementary material:**

The online version of this article (10.1186/s12860-018-0174-z) contains supplementary material, which is available to authorized users.

## Background

Colorectal cancer (CRC) is the second leading cause of cancer death in women. It has been estimated that approximately 1 in 23 women in the United States will develop CRC during their lifetime [1]. The possible risk factors for CRC include age, a diet with lower fiber, a family history of CRC, obesity and smoking [2]. The standard treatment options for CRC include chemotherapy, radiotherapy, and surgery. Despite the continuous advances and improvements in the screening and treatment of CRC [3], the death rate from CRC remains high. Accordingly, identifying effective approaches to control the development and progression of CRC is very important for patients with CRC.

Tanshinone IIA (Tan IIA), which is isolated from the roots of *Salvia miltiorrhiza* Bunge, is currently used to treat patients with cardiovascular disorders, stroke, diabetes and cancers [4–6]. Several studies have reported that Tan IIA exhibits antioxidant, anti-inflammatory, and anticancer activities via multiple effects. In acute stress, Tan IIA alleviates the ischemia-reperfusion injury of the heart [7], liver [8], brain [9] and kidneys [10]. In chronic metabolic disorder, Tan IIA inhibits adipogenic differentiation [11], fatty liver disease [8] and diabetic cardiomyopathy [12]. In addition, the antitumor property of Tan IIA has been well-documented. Tan IIA effectively reduces the progression of ovarian cancer [13], gastric cancer [14], lung tumors [15], and bladder cancer [16]. These findings indicate that Tan IIA could be considered as an adjuvant effective drug to control the progression of human tumors.

At the molecular levels, mitochondria are bioenergetic and biosynthetic organelles that produce enough energy to favor cell metabolism. Mitochondria homeostasis is closely associated with cancer progression and viability. Cancer migration requires sufficient ATP to ensure cell mobility. Cancer protein synthesis and DNA replication are also dependent on mitochondrial function. On the other hand, mitochondrial damage such as mitochondrial oxidative stress and mitochondrial calcium overload can initiate a caspase-9-related mitochondrial apoptotic pathway [17]. Increased mitochondrial apoptosis can induce extensive death of the cancer. Mitochondria also control other apoptotic events, such as endoplasmic reticulum stress, the inflammation response [18], metabolic reprogramming [19], and autophagy [20]. More importantly, mitochondria are the potential target of Tan IIA. In neurons with inflammation damage, Tan IIA mediates mitochondrial oxidative stress [21]. Similarly, in liver cancer [22], prostate cancer [23], and cervical cancer [24], Tan IIA effectively activates mitochondrial apoptosis and promotes cell death. Many researchers have attempted to demonstrate the role of Tan IIA in colorectal cancer death. However, there have been no studies investigating the contribution of Tan IIA in mitochondria-mediated colorectal cancer apoptosis.

Recently, dysregulated mitochondrial dynamics, especially excessive mitochondrial fission, has been found to be associated with mitochondrial apoptosis via multiple effects [25]. Excessive mitochondrial fission produces numerous mitochondrial fragment that contain nonfunctional mitochondria [26]. The mitochondrial fragment with decreased mitochondrial potential and increased membrane permeability could release pro-apoptotic factors (such as Smac) into the cytoplasm/nucleus, inducing caspase-related mitochondrial apoptosis [27]. Mitochondrial fragment contain lower levels of the mitochondrial respiratory complex, impairing energy production [28]. Accordingly, several researchers have proposed that mitochondrial fission is an early hall-marker of mitochondrial apoptosis. In the present study, we asked whether Tan IIA could handle mitochondrial apoptosis by trigging mitochondrial fission.

To this end, mitochondrial fission has been found to be regulated by two signaling pathways: the JNK-Mff axis [29, 30] and the ROCK1-Drp1 pathways [31]. Notably, various pathways seem to be involved in the pathological process of different diseases. For example, in the models of cardiac ischemia reperfusion injury [32] and endometriosis metastasis [33], the JNK-Mff pathway is activated and contributes to the augmentation of mitochondrial fission and cardiomyocyte death. In contrast, in cerebral ischemia reperfusion injury and hyperglycemia-mediated renal damage, mitochondrial fission is primarily activated by the ROCK1-Drp1 pathways [31]. Notably, no study is available to confirm the relationship between ROCK1 and Tan IIA. In contrast, the promotive effect of Tan IIA on the JNK pathways has been well-documented in different disease models [34, 35]. Accordingly, we ask whether Tan IIA could modulate mitochondrial fission via the JNK-Mff pathways. Collectively, the aim of our study was to explore the role of Tan IIA on SW837 colorectal cancer cell viability and investigate whether Tan IIA enhances mitochondrial injury via activating mitochondrial fission in a JNK-Mff pathway-dependent manner.

## Methods

### Cell culture and treatment

In the present study, human rectal cancer cell lines SW837 cells (ATCC® CCL-235™) and SW480 cells (ATCC® CCL-228™) were obtained from the American Type Culture Collection (Manassas, VA, USA). These cells were grown in Dulbecco’s modified Eagle’s medium (DMEM) supplemented with 10% fetal bovine serum (FBS) under 37 °C/5% CO_2_ conditions. To explore the role of Tanshinone IIA (Tan IIA) in SW837 and SW480 cell viability, different doses of Tan IIA (1–20 μM, Sigma-Aldrich, Merck KGaA, cat. no. 568–72-9) were incubated with cells for approximately 12 h. This concentration range was selected based on a previous study [36]. Meanwhile, the cells incubated with PBS were used as the control group. To explore the effects of mitochondrial fission on cell viability, a mitochondrial fission agonist and/or antagonist were used. Mitochondrial division inhibitor 1 (Mdivi1; 10 mM; Sigma-Aldrich; Merck KGaA), an inhibitor of mitochondrial fission, was added into the cell medium for 2 h at 37 °C/5%CO_2_. In contrast, FCCP (5 μm, Selleck Chemicals, Houston, TX, USA), the inducer of mitochondrial fission, was added into the cell medium for approximately 2 h at 37 °C/5%CO_2_ conditions. To explore the role of JNK-Mff pathways in regulating mitochondrial fission, the pathway blocker of JNK was used. SP600125 (25 μM, Selleck Chemicals, Houston, TX, USA), an inhibitor of JNK, was added into the cell medium for 45 min at 37 °C/5%CO_2_. To inhibit the cell apoptosis, cells were pretreated with Z-VAD-FMK (20 μM, Selleck Chemicals) for 1 h.

### EdU assay

Cellular proliferation was detected via the EdU assay using a Click-iT™ Plus EdU Alexa Fluor™ 488 Imaging Kit (Invitrogen, Carlsbad, CA, cat. no. C10637). Briefly, SW837 cells were treated with Tan IIA for 12 h. Then, cells were washed with PBS three times. The EdU solution (50 μM) was incubated with the cells for 2 h at 37 °C/5%CO_2_. Subsequently, PBS was used to wash the cells to remove the free EdU probe. Samples were then fixed with 4% formaldehyde for 30 min, permeabilized with 0.5% Triton X-100 for 10 min and stained with DAPI to label the nucleus for 3 min. After washing with PBS three times, cells were observed under an inverted microscope (BX51; Olympus Corporation, Tokyo, Japan). The percentage of EdU positive cells in at least three randomly selected fields was counted.

### MTT assay and LDH release detection

Cell viability and death were determined using an MTT assay and LDH cytotoxicity assay. The MTT assay was performed using a Vybrant™ MTT Cell Viability Assay (Invitrogen, Carlsbad, CA, cat. no. V13154). The optical density (OD) at 570 nm (reference filter setting was 630 nm) was measured using a plate reader (Spectra Max 190, Molecular Devices, Sunnyvale, CA) according to the manufacturer’s instructions. LDH release detection was analyzed using Pierce™ LDH Cytotoxicity Assay Kit(Invitrogen, Carlsbad, CA, cat. no. 88953). Cells were treated with Tan IIA for 12 h, then the 50 μl of medium solution was transferred to a 96-well plate, which was added to the LDH reaction. The plate was placed at 37 °C/5%CO_2_ for 2 h. To evaluate LDH activity in the medium, the optical density (OD) at 490 nm was recorded.

### Transwell migration assay

Cell migration was estimated via a Transwell assay. Cells treated with Tan IIA were collected using 0.25% trypsin. After being resuspended in fresh DMEM, 1 × 10^3^ cells were added to the upper chamber of 24-well transwell plate. Then, 200 μl of DMEM containing 10% FBS was added into the lower chamber of the 24-well transwell plate. After incubation for 12 h at 37 °C/5%CO_2_, the nonmigrated cells were removed and the migrated cells were fixed with 4% formaldehyde for 30 min followed by staining with 0.05% crystal violet for 15 min at 37 °C/5%CO_2_. At last, the samples were observed under an inverted microscope (BX51; Olympus Corporation, Tokyo, Japan). The percentage of migrated cells in at least three randomly selected fields was counted.

### Mitochondrial ROS measurement

Mitochondrial ROS production was quantified using flow cytometry. First, cells were washed with PBS and then collected using 0.25% trypsin. After being resuspend in fresh DMEM, the MitoSOX™ Red Mitochondrial Superoxide Indicator (Invitrogen, Carlsbad, CA, cat. no. M36008) was used to incubate the cells in the dark at 37 °C/5%CO_2_ for 30 min. Subsequently, PBS was used to wash cells to remove the free ROS probe and then analyzed using a FACSCalibur flow cytometer (BD Biosciences, San Jose, CA).

### Elisa

The concentrations of GOD and GPX were determined using ELISA. Superoxide Dismutase (SOD) Colorimetric Activity Kit (Invitrogen, Carlsbad, CA, cat. no. EIASODC), and the Cellular Glutathione Peroxidase Assay Kit (Beyotime, China, Cat. No. S0056) were used according to the manufacturer’s instructions. Furthermore, the caspase-3 and caspase-9 activities were also measured via ELISA kits. EnzChek™ Caspase-3 Assay Kit (Invitrogen, Carlsbad, CA, cat. no. E13183) and Caspase 9 ELISA Kit (Invitrogen, Carlsbad, CA, cat. no. BMS2025TEN) were performed according to the manufacturer’s instructions.

### JC-1 staining measurement

Mitochondrial potential was observed using the JC-1 probe (eBioscience™ JC-1 Mitochondrial Membrane Potential Dye, Invitrogen, Carlsbad, CA, cat. no. 65–0851-38). In brief, cells were treated with Tan IIA for 12 h and then washed with PBS. Subsequently, the JC-1 probe was used to incubate the cells for 30 min in the dark at 37 °C/5%CO_2_. After washing with PBS, the cells were observed under an inverted microscope (BX51; Olympus Corporation, Tokyo, Japan). The mitochondrial potential was quantified using the red-to-green fluorescence ratio.

### siRNA and transfection

The short interfering RNAs (siRNAs) were synthesized (GenePharma, China) and used for transfection. The sense and antisense strands of the Drp1 siRNA were 5′-GGCAGAGGAAGAAUAUAAATT-3′ and 5′-UUUAUAUUCUUCCUCUGCCTT-3′; negative control were 5′-UUCUCCGAACGUGUCACGUTT-3′ and 5′-ACGUGACACGUUCGGAGAATT-3′. (designed and synthesized by GenePharma Co. Ltd., Shanghai). These siRNAs were transfected into cells with Lipofectamine® 2000 (Invitrogen) following the manufacturer’s instructions. Briefly, a total of 5 × 10^5^ cells were plated in 6-well plates and transfected using 100 pmol siRNA and 5 μL of Lipofectamine® 2000 per well. After 24–48 h of incubation, the cells were harvested for qPCR.

### qPCR

The total RNA was extracted from cells using TRIzol™ Reagent (Invitrogen, Carlsbad, CA, cat. no. 15596018) according to the manufacturer’s instructions. About 1 μg of RNA was reversely transcribed into cDNA with the help of a commercial miScript Reverse Transcription Kit (Qiagen, Valencia, CA, USA). qPCR was performed using miScript PCR Kit (Qiagen) on ABI PRISM 7900HT. The target gene relative expression was calculated using the 2^-ΔΔCt^ method. The primers used in the present study were as follows: ROCK-1: forward primer ACCTGTAACCCAAGGAGATGTG and reverse primer CACAATTGGCAGGAAAGTGG; CDC42, forward primer, ATGCAGACAATTAAGTGTGTTGTTGTGGGCGA, reverse primer, TCATAGCAGCACACACCTGCGGCTCTTCTT; Rac1, forward primer ATGCAGGCCATCAAGTGTGTGG, reverse primer TTACAACAGCAGGCATTTTCTC.

### TUNEL staining and immunofluorescence

A TUNEL assay was performed using the Click-iT™ Plus TUNEL Assay for In Situ Apoptosis Detection (Invitrogen, Carlsbad, CA, cat. no. C10618). In brief, cells were treated with Tan IIA for 12 h and then washed with PBS three times. Subsequently, cells were fixed with 4% formaldehyde for 30 min and permeabilized with 0.5% Triton X-100 for 10 min. Then, the TUNEL probe was incubated with the cells for 4 h at room temperature. After staining with DAPI to label the nucleus for 3 min at room temperature, the samples were observed under an inverted microscope (BX51; Olympus Corporation, Tokyo, Japan). For immunofluorescence, cells were fixed with 4% formaldehyde for 30 min, permeabilized with 0.5% Triton X-100 for 10 min, and blocked with 10% normal fetal bovine serum (Sigma-Aldrich; Merck KGaA). Cells were incubated with primary antibodies at 4 °C overnight, followed by further incubation with secondary antibodies at room temperature for 1 h. After loading with DAPI to label the nucleus, the cells were observed under an inverted microscope (BX51; Olympus Corporation, Tokyo, Japan). The primary antibodies used in the present studies were: Tom20 (1:1000, Abcam, #ab186735), CDK4 (1:1000, Abcam, #ab137675), t-JNK (1:1000; Cell Signaling Technology, #4672), p-JNK (1:1000; Cell Signaling Technology, #9251), Cyclin D1 (1:1000, Abcam, #ab134175), Mff (1:1000, Cell Signaling Technology, #86668), Smac (1:1000, Cell Signaling Technology, #15108), F-actin (1:1000, Abcam, #ab205). Fluorescence intensity was calculated using Image-Pro Plus 6.0 software. Firstly, fluorescence pictures were converted to the grayscale pictures with the help of Image-Pro Plus 6.0 software. Then, fluorescence intensities were separately recorded as the grayscale intensity. Mitochondria in at least 10 cells were observed and the average length of mitochondria was measured to quantify mitochondrial fission under an inverted microscope (BX51; Olympus Corporation, Tokyo, Japan).

### Western blotting

The cell proteins were collected using RIPA Lysis and Extraction Buffer (Invitrogen, Carlsbad, CA, cat. no. 89901). The protein concentration was measured by Pierce™ Rapid Gold BCA Protein Assay Kit (Invitrogen, Carlsbad, CA, cat. no. 53225). Approximately 30–60 μg of protein was loaded with SDS-PAGE gel and then transferred onto a polyvinylidene difluoride (PVDF) membrane on ice. Then, the PVDF membrane was blocked with 5% skim milk in diluted in TBST for 45 min at room temperature. After washing with TBST, the membranes were incubated with primary antibodies. Subsequently, TBST was used to wash the membranes three times, and secondary antibodies were then incubated with the membranes at room temperature for 45 min. The target bands were visualized using enhanced chemiluminescence (ECL, Millipore, CA, USA) technology and the optical densities of the bands were analyzed with Bio-Rad image analysis software (Bio-Rad, Hercules, CA, USA). β-actin and GAPDH was used as a reference to calculate the expression of the target proteins. The primary antibodies used in the present studies were: Drp1 (1:1000, Abcam, #ab56788), Fis1 (1:1000, Abcam, #ab71498), Opa1 (1:1000, Abcam, #ab42364), Mfn2 (1:1000, Abcam, #ab56889), Mff (1:1000, Cell Signaling Technology, #86668), Bcl2 (1:1000, Cell Signaling Technology, #3498), Bax (1:1000, Cell Signaling Technology, #2772), caspase9 (1:1000, Cell Signaling Technology, #9504), pro-caspase3 (1:1000, Abcam, #ab13847), cleaved caspase3 (1:1000, Abcam, #ab49822), c-IAP (1:1000, Cell Signaling Technology, #4952), Bad (:1000; Abcam; #ab90435), PARP (1:1000, Abcam, #ab32064), Smac (1:1000, Cell Signaling Technology, #15108), cadherin (1:1000, Abcam, #ab1416), vimentin (1:1000, Abcam, #ab8978), CDK4 (1:1000, Abcam, #ab137675), t-JNK (1:1000; Cell Signaling Technology, #4672), p-JNK (1:1000; Cell Signaling Technology, #9251), Cyclin D1 (1:1000, Abcam, #ab134175). Experiments were performed in triplicate and repeated three times with similar results.

### Statistics

All results presented in this study were acquired from at least three independent experiments unless indicated specifically. Data were processed and analyzed with PRISM 7.0 and bars display mean ± SEM. The one-way analysis of variance (ANOVA) followed by Tukey’s test was employed for statistical comparison. The statistical significances were calculated as *P* values, and *P* < 0.05 was considered significantly different.

## Results

### Tan IIA induces cell death in a dose-dependent manner

**Fig. 1:**
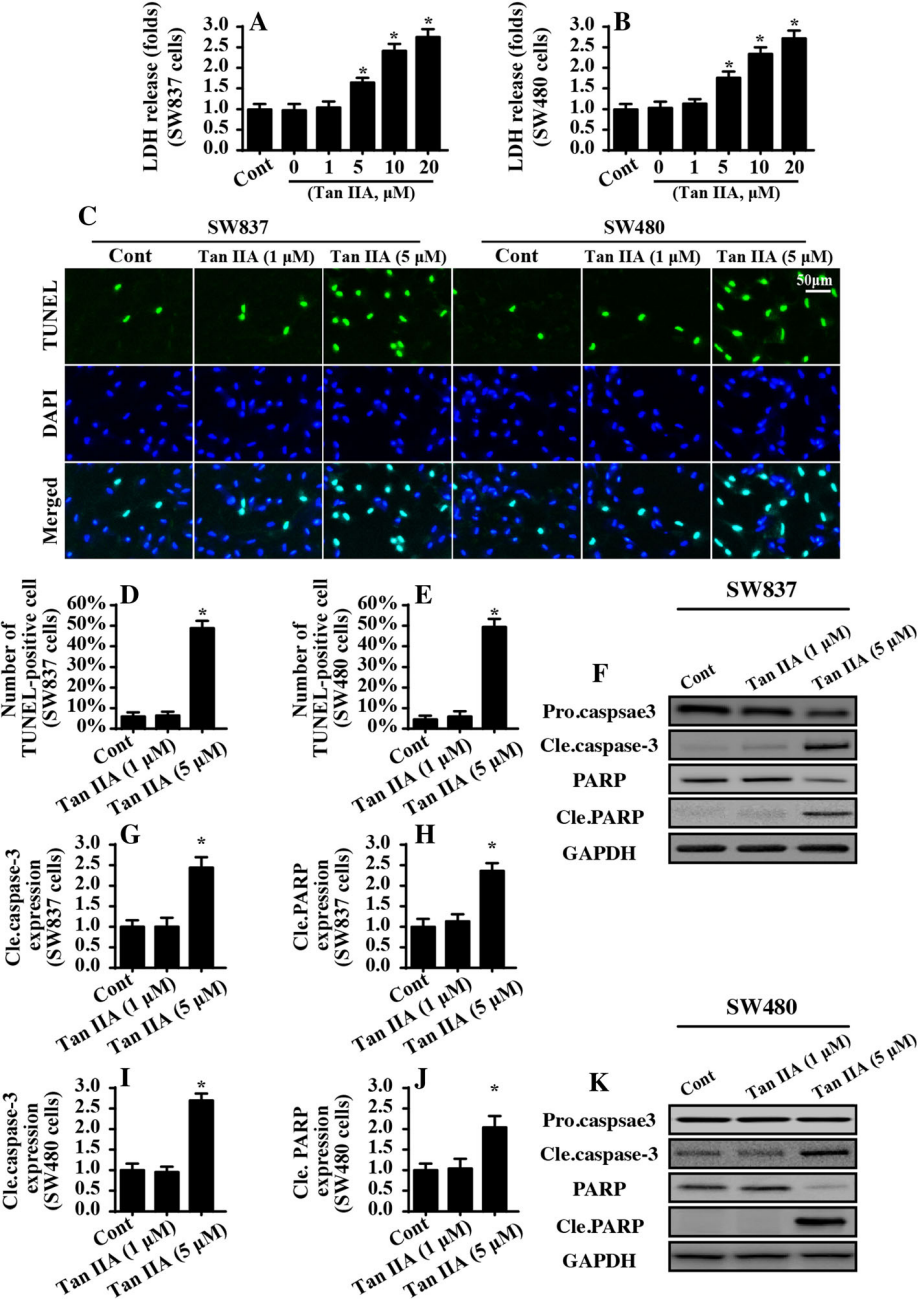
Tan IIA dose-dependently induces cell death. **a** LDH release assay for cell death detection. The relative LDH release was measured in SW837 cells. Different doses of Tan IIA were added into the cell medium for 12 h. **b** LDH release assay in SW480 cells. **c-e** TUNEL staining for cell death. The number of TUNEL-positive cells was counted. Tan IIA at 1 μ M and 5 μ M were incubated with SW837 and SW480 cells for 12 h. **f-k** Western blotting was performed to analyze the protein expression relative to mitochondrial apoptosis. *p < 0.05 vs. control group (cont)

In the present study, different doses of Tan IIA were added and the cell viability of the colorectal cancer cell line SW837 cell was evaluated via an LDH release assay. Compared to the control group, the cell viability was relatively reduced, with an increase in Tan IIA concentration levels (Fig. 1a). To exclude the influence of only one cancer cell line on the results, we also used the SW480 colorectal cancer cell line. As shown in Fig. 1b, Tan IIA also dose-dependently reduced the cell viability of the SW480 cells, as assessed by the LDH-release assays. Similar results were also obtained in MTT assay (Additional file 1: Figure S1A-B). Notably, 1 μ M Tan IIA had no influence on the cell viability and the minimal proapoptotic dose of Tan IIA on SW837 cell and SW480 cell was 5 μ M. Thus, the Tan IIA at 5 μ M was used in the following study.

To further observe the cell death, TUNEL staining was used (Fig. 1c-e). Compared to the control group, 1 μ M Tan IIA had no role in inducing cell death (Fig. 1c-e). However, 5 μ M Tan IIA significantly increased the number of TUNEL-positive cell both in the SW837 and SW480 cells (Fig. 1c-e), reconfirming the proapoptotic role of Tan IIA. Subsequently, Western blotting was used to observe the alterations of protein related to cell death. Compared to the control group, 1 μ M Tan IIA had little influence on Caspase-3 activation, whereas 5 μ M Tan IIA obviously increased the expression of cleaved caspase-3 both in the SW837 cell and SW480 cells (Fig. 1f-k). In response to caspase-3 activation, the expression of cleaved PARP, a substrate of caspase-3, was markedly upregulated in response to Tan IIA treatment both in SW837 cells and SW480 cells (Fig. 1f-k). Altogether, these data indicated that Tan IIA induced cell death in a dose-dependent manner. Notably, no phenotypic differences were observed between the SW837 and SW480 cells in the setting of Tan IIA stress. Thereby, the SW837 cell was used in the following study to explore the molecular mechanisms of Tan IIA.

### Tan IIA treatment mediates proliferation arrest and migration inhibition

**Fig. 2:**
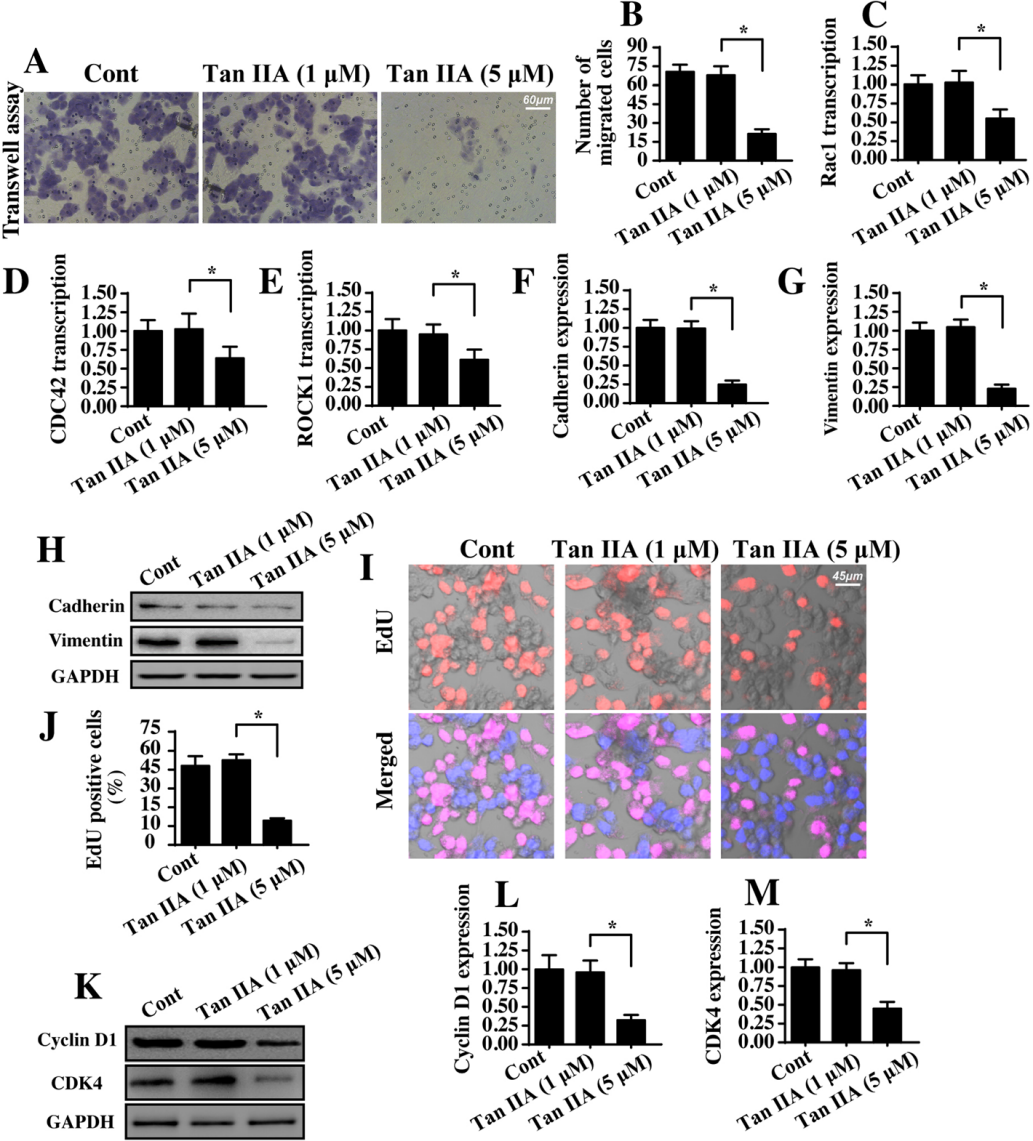
Tan IIA inhibits cancer cell proliferation and migration. **a-b** The transwell assay was used to analyze the cell mobility. The number of migrated cells was recorded. Tan IIA at 1 μ M and 5 μ M were incubated with SW837 cells for 12 h. **c-e** qPCR assay for metastatic gene transcription. Compared to the control group, Tan IIA significantly increased the transcription of Rac1, CDC42, ROCK1. **f-h** The expression of adhesive factors were determined via western blotting. **i-j** EdU staining for cell proliferation. The number of EdU-positive cells was recorded. **k-m** Western blotting analysis for proliferation-related proteins. SW837 cells were treated with 1 μ M or 5 μ M Tan II for 12 h. *p < 0.05

Next, experiments were performed to verify the role of Tan IIA in cell migration and proliferation. First, the transwell assay was conducted in SW837 cells with Tan II treatment. Then, the number of migrated cells was calculated. As shown in Fig. 2a-b, Tan IIA treatment markedly increased the ratio of migrated cells when compared to the control group. These results identified the inhibitory effects of Tan IIA on cell migration. To determine the mechanism by which Tan IIA repressed cell mobility, we evaluated the transcription of metastatic gene. As shown in Fig. 2c-e, compared to the control group, Tan IIA treatment reduced the mRNA transcription of Rac1, CDC42 and ROCK1 in SW837 cells. Besides, we also analyzed the proteins expression of adhensive factors. Compared to the control group (Fig. 2f-h), Tan IIA treatment obviously repressed the expression of cadherin and vimentin, reconfirming the inhibitory effect of Tan IIA on SW837 cell migration.

With respect to cancer cell proliferation, EdU staining was used. As shown in Fig. 2i-j, ~ 50% of the SW837 cell was EdU positive, and this ratio was reduced to ~ 10% under Tan IIA treatment, highlighting the antiproliferative capacity of Tan IIA. The reduction in EdU positive cells demonstrates cell cycle arrest. Accordingly, Western blotting was performed to analyze the protein expression of cell cycle agents, such as CDK4 and Cyclin D1. As shown in Fig. 2k-m, abundant expression of CDK4 and Cyclin D1 were noted in the control group in SW837 cells. However, Tan IIA treatment drastically repressed the levels of CDK4 and Cyclin D1. Altogether, our data suggested that Tan IIA inhibited proliferation and migration in SW837 cells.

### Tan IIA triggers mitochondrial damage

**Fig. 3:**
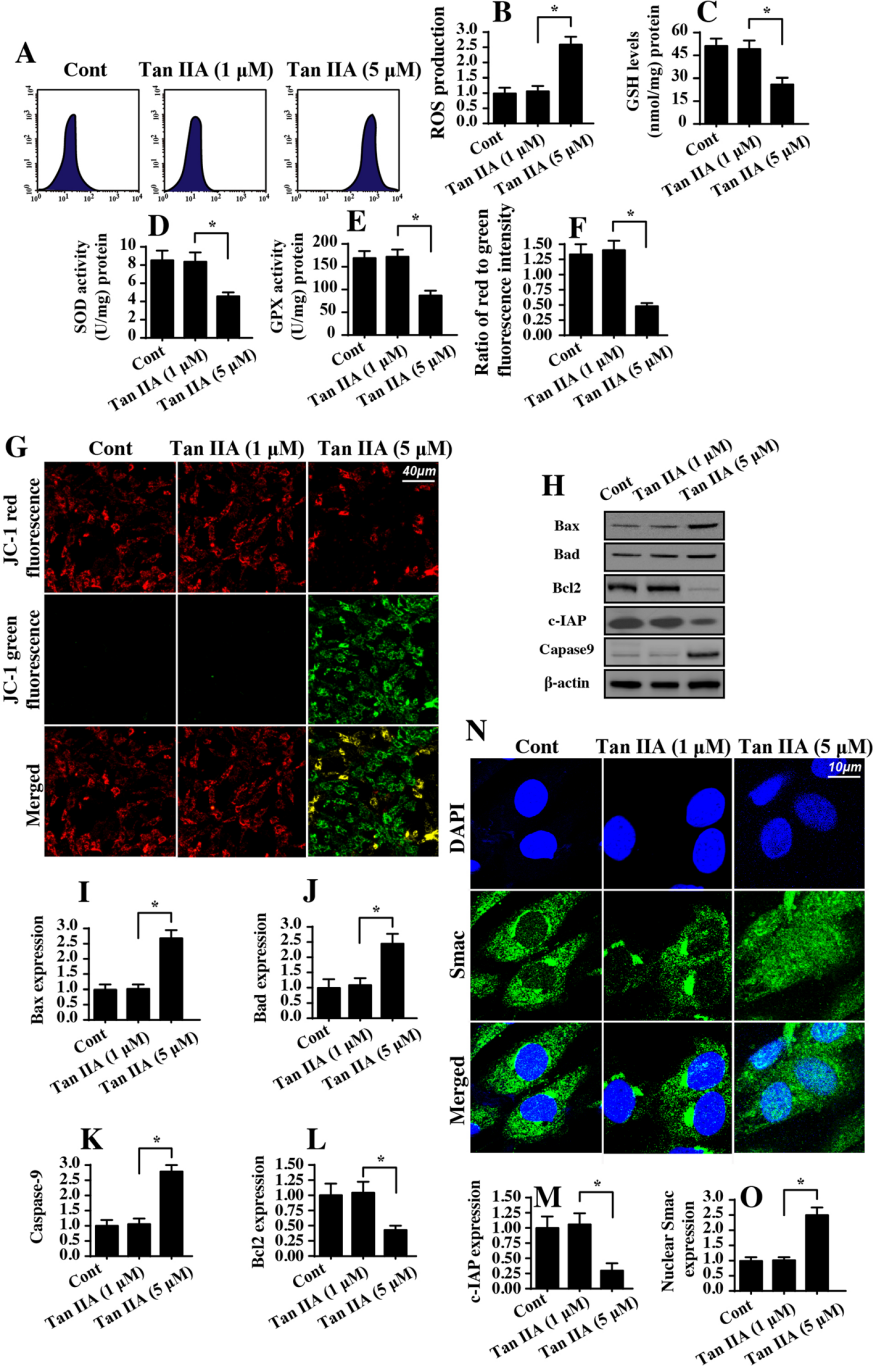
Tan IIA promotes mitochondrial injury. **a-b** Mitochondrial ROS production was measured via flow cytometry using MitoSOX™ Red Mitochondrial Superoxide Indicator. Tan IIA at 1 μ M and 5 μ M were incubated with SW837 cells for 12 h. **c-e** The antioxidant factors, including GSH, SOD and GPX were quantified via ELISA in SW837 cells treated with Tan IIA. **f-g** Mitochondrial potential was observed using JC-1 staining and quantified by evaluating the ratio of red-to-green fluorescence intensity. **h-m** The alterations of mitochondrial proapoptotic and antiapoptotic proteins were detected using Western blotting. **n-o** Immunofluorescence assay for Smac. Blue fluorescence is the DAPI probe, which stained the nucleus. *p < 0.05

Mitochondrial homeostasis is closely associated with cell viability and mobility. Accordingly, we explored the role of Tan IIA in mitochondrial function in SW837 cells. First, mitochondrial ROS was detected via flow cytometry. Compared to the control group, mitochondrial ROS was significantly increased in response to Tan IIA treatment (Fig. 3a-b). This effect was followed by a decrease in the concentration of cellular antioxidant factors including GSH, GPX and SOD (Fig. 3c-d). This information indicated that Tan IIA promoted a cell redox imbalance via mitochondrial oxidative stress. As a consequence of mitochondrial oxidative stress, we found that the mitochondrial membrane potential collapsed in response to Tan IIA treatment, as evidenced by higher green and lower red JC-1 fluorescence (Fig. 3f-g). To quantify the alterations of mitochondrial membrane potential, we evaluated the ratio of red-to-green fluorescence intensity. As shown in Fig. 3f-g, compared to the control group, the ratio of red/green fluorescence was significantly reduced in the Tan IIA-treated cells. The reduction in mitochondria potential is an early event in mitochondrial apoptosis. Based on this, we explored the alterations of mitochondrial apoptotic proteins. As shown in Fig. 3h-m, the levels of mitochondrial proapoptotic factors such as Bax and Bad were significantly upregulated in Tan IIA-treated cells. In contrast, the expression of mitochondrial antiapoptotic proteins such as c-IAP and Bcl2 were relatively downregulated in response to Tan IIA stress (Fig. 3h-m). This effect was also followed by an increase in caspase-9 expression, the critical executor feature of mitochondrial apoptosis (Fig. 3h-m). Besides, we confirmed that mitochondrial Smac was translocated into the cytoplasm/nucleus in the presence of Tan IIA (Fig. 3n-o). To quantify the Smac liberation, we evaluated the expression of nuclear Smac. As shown in Fig. 3n-o, Tan IIA treatment largely elevated the levels of nuclear Smac when compared to the control group. Altogether, our results indicated that Tan IIA triggered mitochondrial damage and activated caspase-9-related mitochondrial apoptosis in SW837 cells.

### Mitochondrial fission is activated by tan IIA in SW837 cells

**Fig. 4:**
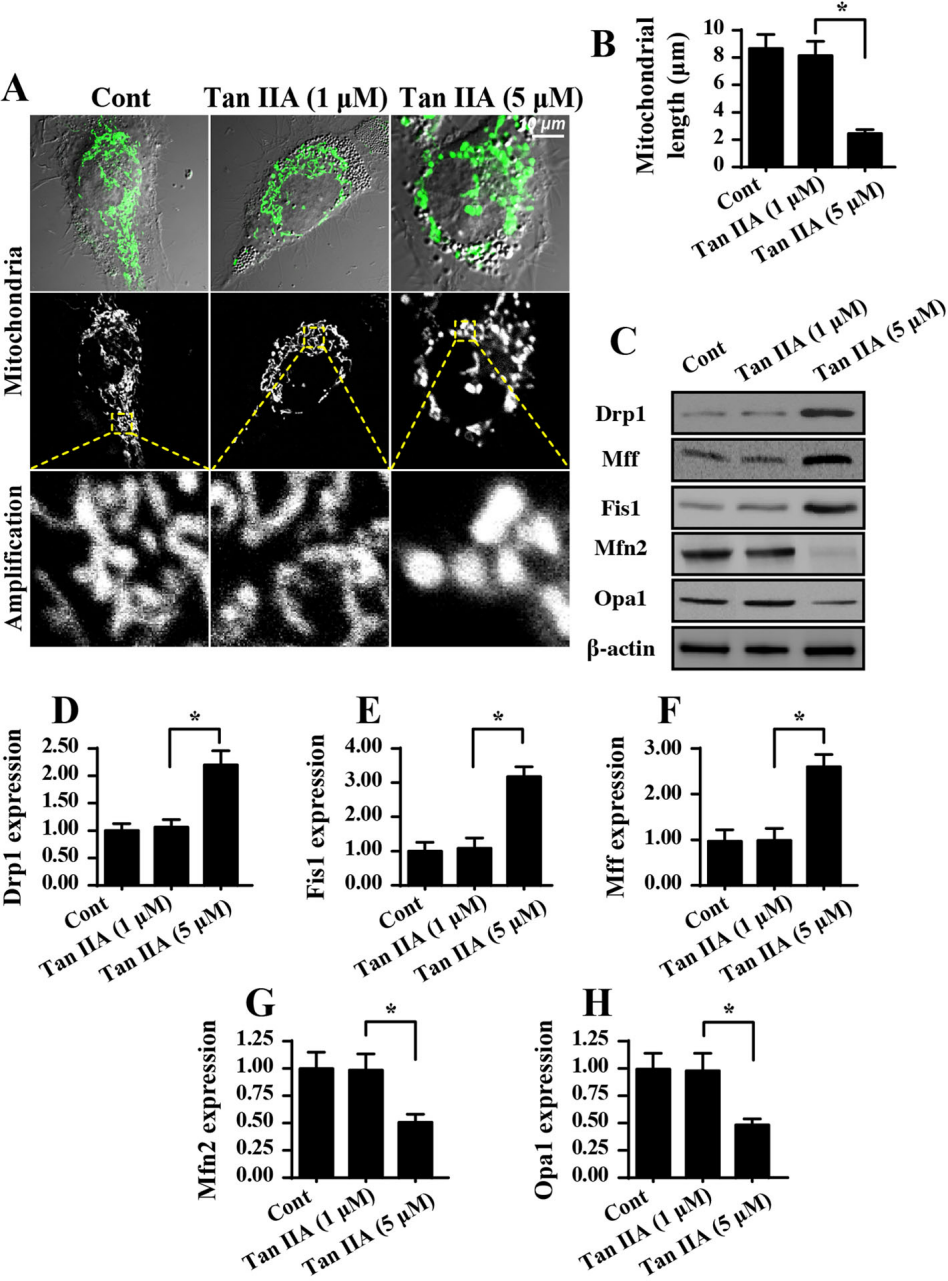
Tan IIA activates mitochondrial fission in SW837 cells. **a-b** Immunofluorescence assay for mitochondria using the mitochondrial specific antibody Tom-20. The mitochondrial fragment was counted to quantify the mitochondrial fission. **c-h** Western blotting for fission-related proteins. Pro-fission factors such as Drp1, Fis1 and Mff were increased in response to Tan IIA treatment. In contrast, anti-fission factors including Mfn2 and Opa1 were also downregulated in Tan IIA-treated cells. *p < 0.05

Recent studies have identified mitochondrial fission as an upstream regulator for mitochondrial homeostasis, especially mitochondrial damage and mitochondrial apoptosis [37]. Considering the promotive role of Tan IIA in mitochondrial injury, we asked whether Tan IIA-mediated mitochondrial malfunction by activating mitochondrial fission. First, an immunofluorescence assay for mitochondria was performed using the mitochondrial specific antibody Tom-20. Then, the mitochondrial morphology was observed. In the control group, mitochondria formed a network exhibiting longer and inter-connective morphological character (Fig. 4a). However, after Tan IIA treatment, the mitochondria divided into several fragments with shorter and rounder morphological features. This result indicated that Tan II possibly promoted mitochondrial fission in SW837 cells. Subsequently, the average mitochondrial length was counted, and these data demonstrated that Tan IIA treatment shortened the overall mitochondrial length (Fig. 4b).

This finding was further supported by analyzing the proteins related to mitochondrial fission. The levels of pro-fission factors such as Drp1, Mff and Fis1 were significantly increased in response to Tan IIA treatment (Fig. 4c-h). However, the expression of anti-fission proteins including Mfn2 and Opa1 were correspondingly downregulated in the Tan IIA-treated cells (Fig. 4c-h). This information further supported the functional importance of Tan IIA in trigging mitochondrial fission in SW837 cells.

### Mitochondrial fission is required for tan IIA-mediated mitochondrial damage

**Fig. 5:**
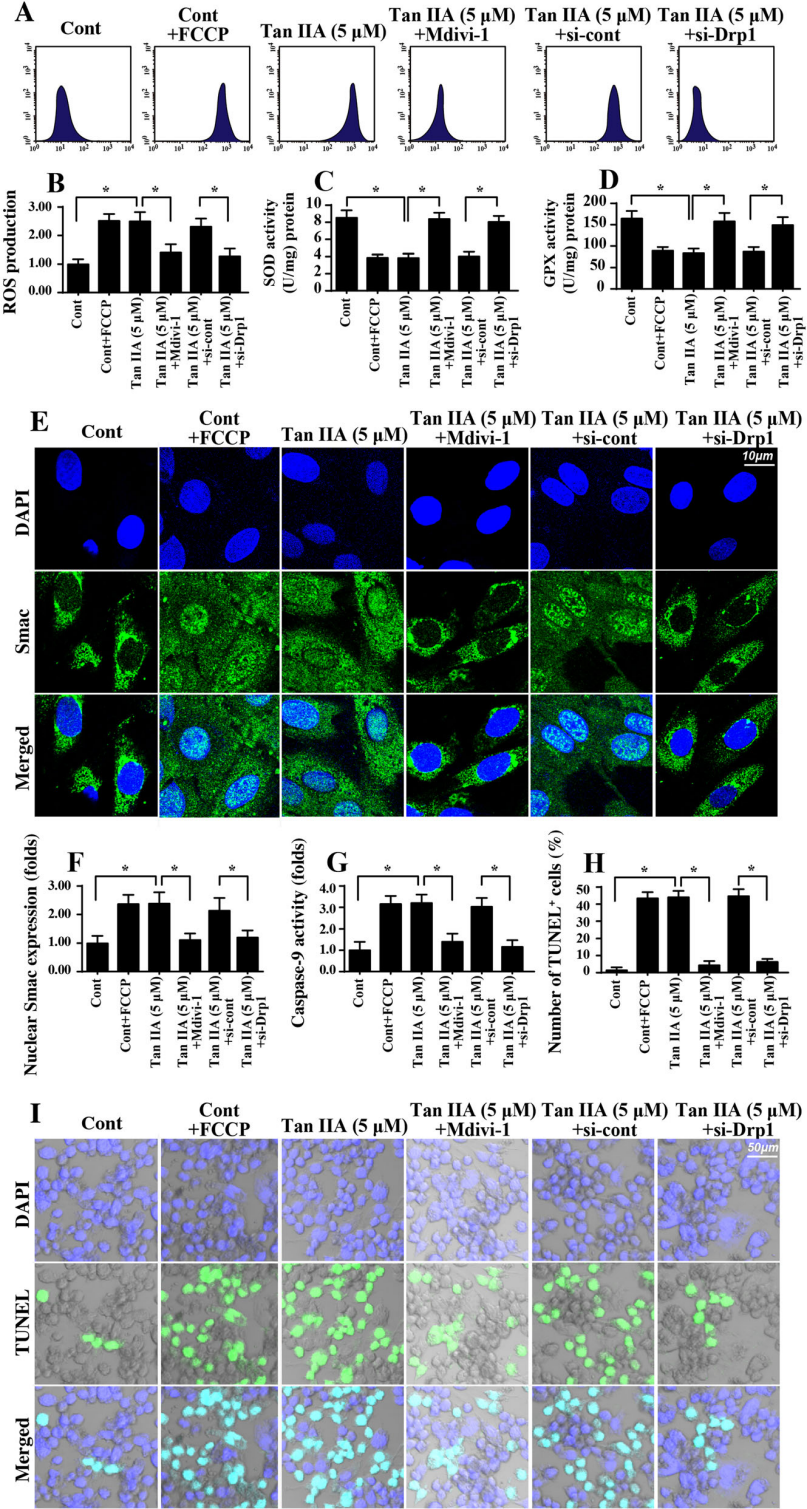
Tan IIA-mediated mitochondrial fission triggers mitochondrial damage. **a-b** Mitochondrial ROS production was measured via flow cytometry. Mdivi-1 and Drp1 siRNA were used to prevent fission, which was used as the loss-of-function assay for mitochondrial fission. The mitochondrial fission agonist (FCCP) was added to the control group to activate mitochondrial fission, and this was used to mimic the effects of Tan IIA. **c-d** Antioxidant factors, including SOD and GPX, were quantified via ELISA in SW837 cells treated with Tan IIA. **e-f** Immunofluorescence assay for Smac. Blue fluorescence is from the DAPI probe, which stains the nucleus. **g** Caspase-9 activity was determined via ELISA. Mdivi-1 and Drp1 siRNA were used to prevent fission, which was used as the loss-of-function assay for mitochondrial fission. The mitochondrial fission agonist (FCCP) was added to the control group to activate mitochondrial fission, and this was used to mimic the effects of Tan IIA. **h-i** TUNEL staining to assess cell death. The number of TUNEL-positive cell was counted. *p < 0.05

Next, experiments were performed to verify the contribution of mitochondrial fission in Tan IIA-mediated mitochondrial damage and cell apoptosis in SW837 cells. We added the mitochondrial fission agonist (FCCP) to the control group to activate mitochondrial fission, which was used to mimic the effects of Tan IIA. Moreover, Mdivi-1 and Drp1 siRNA were used to prevent mitochondrial fission. Then, mitochondrial function was estimated again. The knockdown efficiency of Drp1 siRNA was shown in Additional file 1: Figure S1C-D. As shown in Fig. 5a-b, mitochondrial ROS was significantly increased in Tan IIA-treated cells, and this effect was similar to the results via activation of mitochondrial fission using FCCP. However, Tan IIA-mediated mitochondrial ROS overloading was negated by Mdivi-1 and Drp1 siRNA (Fig. 5a-b), suggesting that a blockade of fission protected mitochondria against Tan IIA-exerted oxidative stress. As a consequence of the cell redox balance, Tan IIA-mediated the downregulation of antioxidant factors and was also reversed by Mdivi-1 in SW837 cells (Fig. 5c-d). Altogether, our data confirmed the necessary role played by mitochondrial fission in regulating Tan IIA-induced mitochondrial oxidative stress. Furthermore, mitochondrial Smac liberation was drastically augmented by Tan IIA, and this effect was mostly repressed by Mdivi-1 and Drp1 siRNA (Fig. 5e-f). Along with a fall in Smac translocation, the Tan IIA-mediated caspase-9 activation was also largely inhibited by Mdivi-1 and Drp1 siRNA (Fig. 5g). The above data illustrated that Tan IIA mediated mitochondrial damage in a manner dependent on mitochondrial fission. To this end, a TUNEL assay was used to observe the cell death in response to Tan IIA and/or mitochondrial fission inhibition. Compared to the control group, Tan IIA stress and FCCP treatment significantly elevated the number of TUNEL-positive cells (Fig. 5h-i). However, the proapoptotic effects of Tan IIA on SW837 cells were abolished via a blockade of mitochondrial fission using Mdivi-1 and Drp1 siRNA (Fig. 5h-i). Altogether, our results demonstrated that Tan IIA induced mitochondrial fission to cause mitochondrial damage and cell apoptosis in SW837 cells.

### Mitochondrial fission promotes proliferation arrest and migration inhibition

**Fig. 6:**
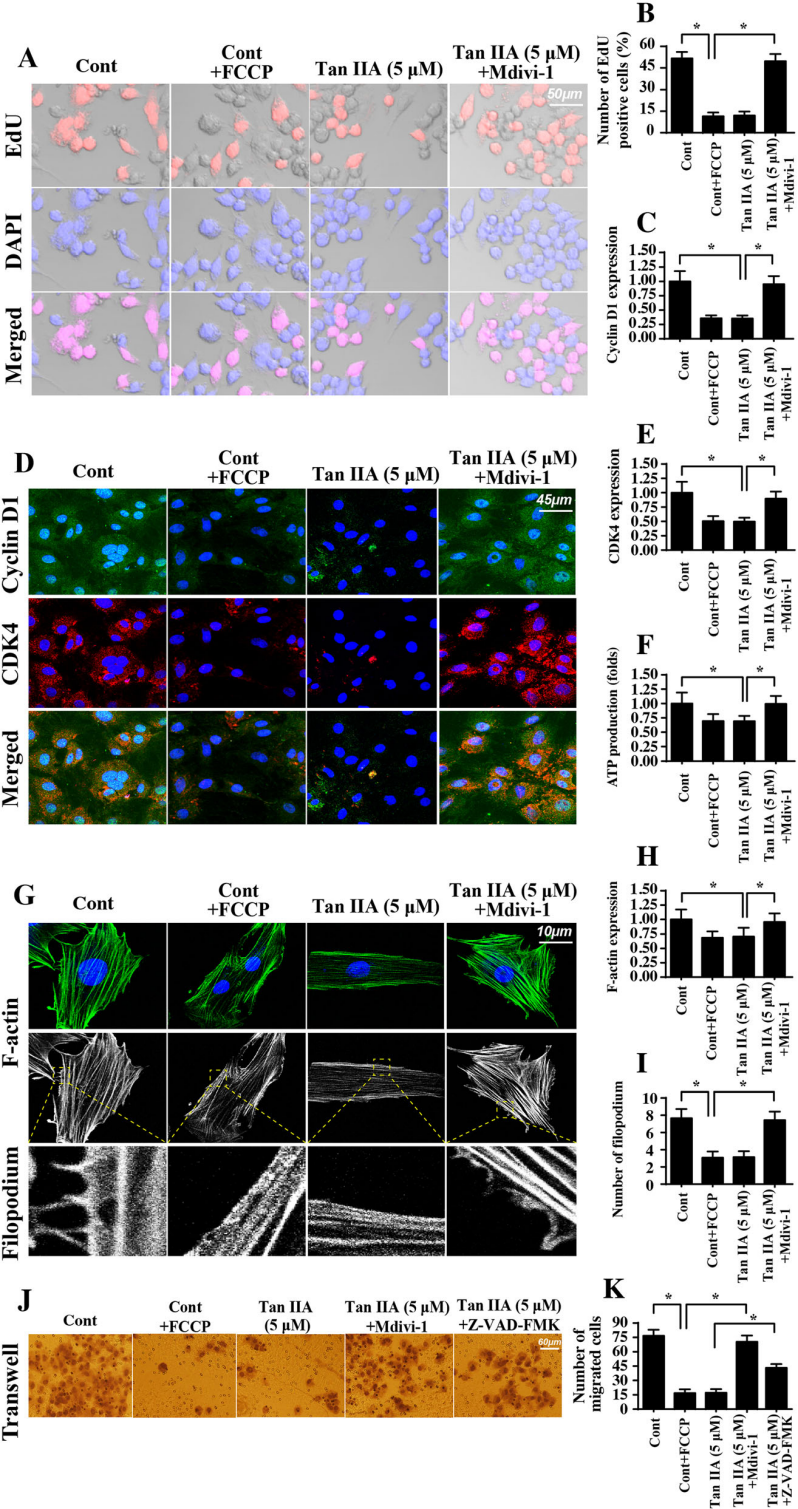
Mitochondrial fission is implicated in Tan IIA-modulated SW837 cell migration and proliferation. **a-b** EdU staining for SW837 cells. The number of EdU positive cells was recorded. Mdivi-1, an antagonist of mitochondrial fission was administered into Tan IIA-treated cells to prevent fission, and this was used as a loss-of-function assay for mitochondrial fission. Mitochondrial fission agonist (FCCP) was added into the control group to activate mitochondrial fission, which was used to mimic the effects of Tan IIA. **c-e** Immunofluorescence analysis for the proliferation-related proteins. Mdivi-1 and FCCP were administered to perform the loss- and gain-of-function assays to assess mitochondrial fission, respectively. **f.** ATP production was detected via ELISA. **g-i** Immunofluorescence assay for F-actin and filopodium. The length of filopodium was recorded. Mdivi-1 and FCCP were administered to perform the loss- and gain-of-function assays for mitochondrial fission, respectively. **j-k** Transwell assay in the presence of Mdivi-1 and Z-VAD-FMK. *p < 0.05

We also wanted to know whether mitochondrial fission was also implicated in Tan IIA-mediated proliferation arrest and migration impairment in SW837 cells. To address this question, loss- and gain-of-function assays for mitochondrial fission were performed using Mdivi-1 and FCCP, respectively. To evaluate cell proliferation, an EdU assay was conducted. The results shown in Fig. 6a-b indicated that either Tan IIA treatment or FCCP supplementation significantly reduced the ratio of EdU-positive cells. Interestingly, Mdivi-1 application drastically reversed the percentage of EdU-positive cells (Fig. 6a-b), suggesting that the antiproliferation property of Tan IIA is achieved by activating mitochondrial fission. Furthermore, the protein expression of Cyclin D1 and CDK4 were both downregulated in Tan IIA-treated cells and were reversed to near-normal levels with Mdivi-1 treatment (Fig. 6c-d), reconfirming the idea that mitochondrial fission is necessary for Tan IIA-mediated proliferation termination.

With respect to cell migration, we first estimated the ATP production, since a sufficient ATP content is a perquisite for cell mobilization. As shown in Fig. 6f, compared to the control group, the concentration of ATP was unfortunately reduced in response to Tan IIA or FCCP treatment. However, Mdivi-1 treatment would reverse the ATP production in Tan IIA-treated cells (Fig. 6f). Furthermore, cell migration is highly dependent on F-actin synthesis and filopodium formation. With the assistance of immunofluorescence, we found that F-actin expression was lower in Tan IIA-treated or FCCP-incubated cells than in the control cells (Fig. 6g-h). Interestingly, Mdivi-1 treatment significantly reversed the expression of F-actin in SW837 cells despite treatment with Tan IIA (Fig. 6g-h). In addition, we measured the length of filopodium, which is composed of F-actin. Compared to the control group, the length of filopodium was reduced in response to Tan IIA treatment or FCCP administration (Fig. 6i). However, blockade of mitochondrial fission using Mdivi-1 could reverse the length of filopodium in the presence of Tan IIA (Fig. 6i). Besides, we also found that Tan IIA-mediated migration inhibition could be reversed by Mdivi-1 treatment. Interestingly, caspase inhibitor (Z-VAD-FMK) could partly increase the number of migrated cells in the presence of Tan IIA (Fig. 6j-k), suggesting that apoptosis partly contributed to the Tan IIA-mediated cancer migration inhibition. Altogether, our results confirmed that SW837 cell migration was regulated by Tan IIA via mitochondrial fission.

### Tan IIA regulates mitochondrial fission via the JNK-Mff axis

**Fig. 7:**
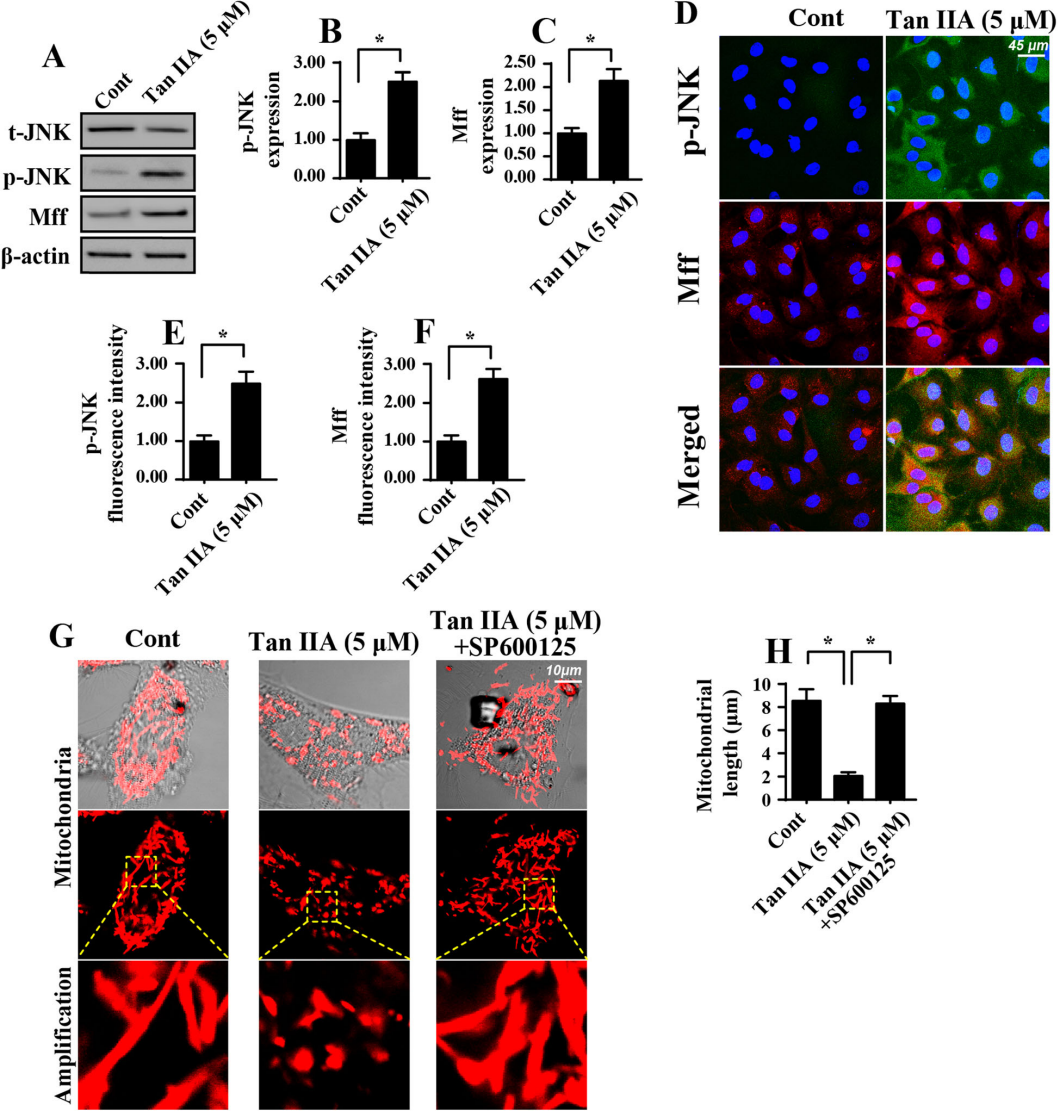
Tan IIA activates mitochondrial fission via the JNK-Mff pathways. **a-c** Western blotting was conducted to verify the alteration of JNK phosphorylation and Mff expression. **d-f** Immunofluorescence assays for JNK phosphorylation and Mff expression. Tan IIA treatment significantly increased the expression of p-JNK and Mff. **g-h** Mitochondrial fission was observed via immunofluorescence using the mitochondrial-specific antibody Tom-20. The average length of the mitochondria was recorded. SP600125, an inhibitor of JNK, was added to the Tan IIA-treated cells to prevent JNK activation. *p < 0.05

To this end, we determined the molecular basis by which Tan IIA regulated mitochondrial fission in SW837 cells. Previous studies have suggested that two signaling pathways, including the JNK-Mff axis and ROCK1-Drp1 cascade [32], are associated with mitochondrial fission activation. Considering the links between the Tan IIA and JNK-Mff pathways, we hypothesized that Tan IIA elevated mitochondrial fission by activating JNK-Mff pathways. Western blotting analysis shown in Fig. 7a-c demonstrated that JNK phosphorylation and Mff expression was significantly increased in Tan IIA-treated cells when compared to the control group. This finding was further validated via an immunofluorescence assay. The fluorescence intensity of p-JNK and Mff were both increased in response to Tan IIA treatment (Fig. 7d-f). Therefore, the above data substantiated the sufficiency of Tan IIA in activating the JNK-Mff axis. Furthermore, to explain whether JNK-Mff pathways were implicated in Tan IIA-mediated mitochondrial fission, a pathway blocker was used in the Tan IIA-treated cells. Subsequently, the mitochondrial morphology was observed again. As shown in Fig. 7g-h, compared to the control group, Tan IIA treatment forced the mitochondria to divide into several fragments, and this effect was negated by JNK antagonists. Altogether, our results indicated that Tan IIA regulated mitochondrial fission via JNK-Mff pathways.

## Discussion

In the present study, we provided evidence for the regulatory role of Tan IIA in SW837 cells, with a focus on the mitochondrial fission and JNK-Mff pathways. We demonstrated that Tan IIA dose-dependently increased the apoptotic index of SW837 cells. Furthermore, Tan IIA also repressed SW837 cell migration and proliferation. The molecular investigation illustrated that Tan IIA mediated mitochondrial damage in SW837 cells via activating mitochondrial fission. Excessive mitochondrial fission promoted mitochondrial ROS production, reduced mitochondrial membrane potential, suppressed ATP generation, induced mitochondrial Smac translocation into cytoplasm/nucleus, and activated caspase-9-related mitochondrial apoptosis. However, a blockade of mitochondrial fission could abrogate the inhibitory effects of Tan IIA on SW837 cell survival, migration and proliferation. To this end, we confirmed that Tan IIA modulated mitochondrial fission via the JNK-Mff pathways. The inhibition of the JNK-Mff axis abolished the promotive action of Tan IIA on mitochondrial fission. Overall, our results first demonstrated that Tan IIA induced colorectal cancer line apoptosis, migration inhibition and proliferation arrest via activating mitochondrial fission in a manner dependent on JNK-Mff pathways. To the best of our knowledge, this is the first study to verify the molecular mechanisms of Tan IIA on mitochondrial fission on one hand. On the other hand, our study identifies mitochondrial fission as a potential target to influence the phenotype of colorectal cancer, and therefore, strategies to enhance mitochondrial fission would be considered as effective approaches to treat colorectal cancer in the clinical practice.

Tan IIA, an active ingredient extracted from Danshen, has been tested in several diseases, such as angina, chronic fatty liver disease, diabetes, nephritis and tumors [4–6]. Ample evidence has confirmed its antioxidative, anti-inflammatory, antineoplastic, and anticancer properties. In addition, numerous studies have attempted to use Tan IIA as an adjunct drug to retard the progression of cancer via promoting cancer death and impairing cell proliferation. Its tumor-suppressive effects have been characterized in different kinds of cancers such as in liver cancer [22], prostate cancer [23], cervical cancer [24], and colorectal cancer [36]. Our study is in agreement with the previous findings. We demonstrated that Tan IIA mediated SW837 cell death, migration inhibition and proliferation arrest. This information provides evidence to apply Tan IIA, a kind of traditional Chinese medicine, to treat cancer in the colon and rectum. Notably, more clinical evidence will be required to support our findings. Notably, we also noted the different percentages of cell viability in MTT assay and TUNEL staining. This may be attributable to residual mitochondrial activity at the early stages of apoptosis in response to Tan IIA.

At the molecular levels, we found that Tan IIA mediated SW837 cell stress by inducing mitochondrial injury. Tan IIA treatment was associated with decreased mitochondrial potential, increased mitochondrial ROS production, repressed ATP production, augmented Smac leakage, and activated caspase-9 mitochondrial apoptosis pathways. This finding was similar to that of several previous studies, which identified mitochondria as a potential target of Tan IIA in various cancers such as oral cancer [38], non-small cell lung cancer [39], and bladder cancer [40]. Interestingly, several previous studies found that Tan IIA treatment could reduce ROS production in the acute renal damage [41] and endothelial oxidative stress models [42, 43], suggesting that Tan IIA has the ability to attenuate ROS overloading. Notably, in several cancer cells, such as colorectal cancer [36] and lung cancer [44], Tan IIA could increase ROS generation leading to cancer cell oxidative stress. The above data highlight that the regulatory role of Tan IIA in redox balance seems to dependent on the cell type.

In the present study, we for the first time demonstrated that Tan IIA mediated mitochondrial injury via mitochondrial fission. Although the deleterious effects of mitochondrial fission in cancer viability have been well-characterized, the functional role of mitochondrial fission in colorectal cancer has not been explored. Thus our results have helped to fill this gap. We demonstrated that mitochondrial fission promoted the death of SW837 cells. Increased mitochondrial fission repressed the mitochondrial potential, elevating caspase-9 activity and evoking cell death. Furthermore, activated mitochondrial fission also reduced F-actin and impaired the formation of filopodium, finally blunting cell migration. Notably, the pro-apoptotic effect of Tan IIA may contribute to the migration inhibition of SW480 cells because inhibition of apoptosis partly improved cell migration. Interestingly, we also observed that the levels of adhensive factors (cadherin and vimenti) were downregulated in response to Tan IIA treatment, suggesting that Tan IIA may directly control the cancer migration. However, more researches are required to further figure out the primary mechanism of Tan IIA-mediated cancer migration inhibition.

Finally, we found that Tan IIA modulated mitochondrial fission by repressing JNK-Mff pathways. In fact, mitochondrial fission is primarily regulated by two signaling pathways. One is the ROCK1-Drp1 axis [31] and the other is the JNK-Mff cascade [29, 30, 45]. Notably, these two signaling pathways have been reported in different disease models. In the present study, our data demonstrated that Tan IIA promoted JNK phosphorylation. The latter upregulated the levels of Mff. However, blockade of JNK-Mff pathways would abrogate the permissive role of Tan IIA in mitochondrial fission. These results indicated that Tan IIA modulated mitochondrial fission, at least when it occurred via the JNK-Mff axis. However, whether ROCK1-Drp1 is required for Tan IIA-mediated mitochondrial function remains unknown, and more studies are thus required to provide an answer to this question.

## Conclusion

Collectively, our results have provided a novel piece of evidence to explain the anti-tumor effects of Tan IIA on colorectal cancer phenotypes involving survival, migration and proliferation. Tan IIA treatment mediated mitochondrial injury via activating JNK-Mff-mitochondrial fission signaling pathways. This finding identifies mitochondrial fission as a new potential target to control cancer viability and suggests that Tan IIA is an effective drug to treat colorectal cancer through mitochondrial fission.

## Additional file

Additional file 1: **Figure S1.** A-B. MTT assay in SW837 cells and SW480 cells. Different doses of Tan IIA was used to incubate with cancer cells and then cell viability was determined via MTT assay. C-D. The expression of Drp1 in response to Drp1 siRNA. *p < 0.05 vs. control group; #*p* < 0.05 vs. Tan IIA + si-cont group. (DOCX 133 kb)

## Abbreviations

CRC: Colorectal cancer
Mff: Mitochondrial fission factor
Tan IIA: Tanshinone IIA
