# Supplementary material for: Tanshinone IIA reduces SW837 colorectal cancer cell viability via the promotion of mitochondrial fission by activating JNK-Mff signaling pathways
Source: BMC Cell Biol. 2018 Sep 25;19:21. doi: 10.1186/s12860-018-0174-z (PMC6157045; doi:10.1186/s12860-018-0174-z)
Supplement: Supplementary file 1 — Figure S1. A-B. MTT assay in SW837 cells and SW480 cells. Different doses of Tan IIA was used to incubate with cancer cells and then cell viability was determined via MTT assay. C-D. The expression of Drp1 in response to Drp1 siRNA. *p < 0.05 vs. control group; #p < 0.05 vs. Tan IIA + si-cont group. (DOCX 133 kb) [file 12860_2018_174_MOESM1_ESM.docx]

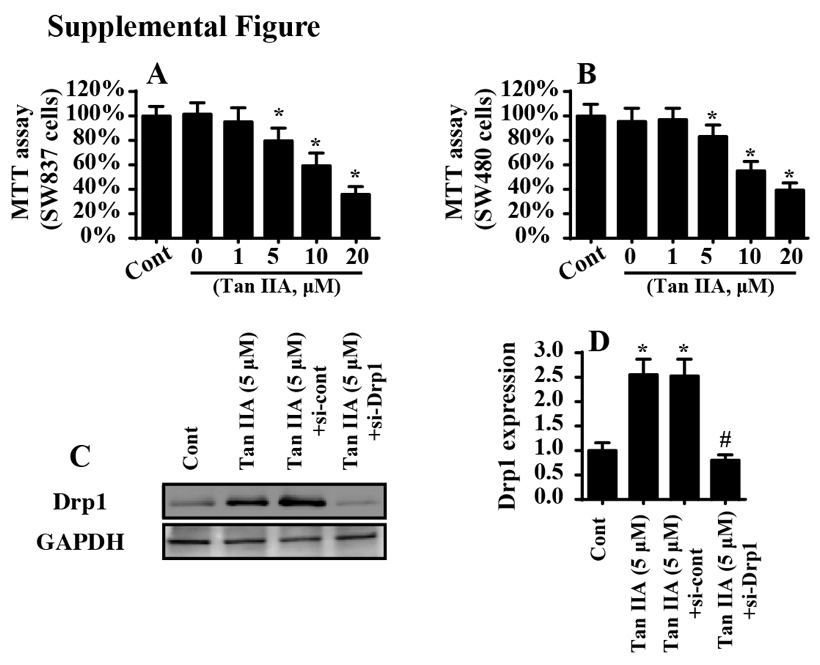


**Figure S1** A-B. MTT assay in SW837 cells and SW480 cells. Different doses of Tan IIA was used to incubate with cancer cells and then cell viability was determined via MTT assay. C-D. The expression of Drp1 in response to Drp1 siRNA. **p*<0.05 *vs.* control group; #*p*<0.05 *vs.* Tan IIA+si-cont group.
